# PEO-PPO-PEO Tri-Block Copolymers for Gene Delivery Applications in Human Regenerative Medicine—An Overview

**DOI:** 10.3390/ijms19030775

**Published:** 2018-03-08

**Authors:** Ana Rey-Rico, Magali Cucchiarini

**Affiliations:** 1 Center of Experimental Orthopaedics, Saarland University Medical Center, Kirrbergerstr. Bldg 37, D-66421 Homburg/Saar, Germany; magali.madry@uks.eu; 2 Centro de Investigacións Científicas Avanzadas (CICA), Universidade da Coruña, Campus de A Coruña, 15071 A Coruña, Spain

**Keywords:** PEO-PPO-PEO copolymers, nonviral vectors, viral vectors, gene transfer

## Abstract

Lineal (poloxamers or Pluronic^®^) or X-shaped (poloxamines or Tetronic^®^) amphiphilic tri-block copolymers of poly(ethylene oxide) and poly(propylene oxide) (PEO-PPO-PEO) have been broadly explored for controlled drug delivery in different regenerative medicine approaches. The ability of these copolymers to self-assemble as micelles and to undergo sol-to-gel transitions upon heating has endowed the denomination of “smart” or “intelligent” systems. The use of PEO-PPO-PEO copolymers as gene delivery systems is a powerful emerging strategy to improve the performance of classical gene transfer vectors. This review summarizes the state of art of the application of PEO-PPO-PEO copolymers in both nonviral and viral gene transfer approaches and their potential as gene delivery systems in different regenerative medicine approaches.

## 1. PEO-PPO-PEO Tri-Block Copolymers

**Figure 1:**
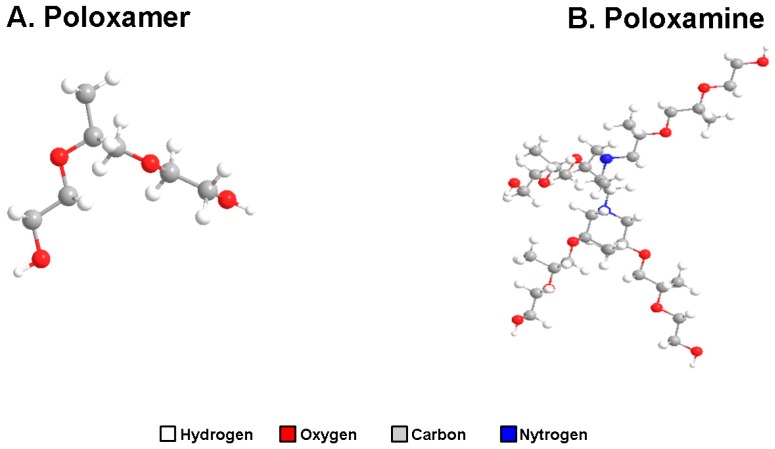
Structure of poloxamers (**A**) and poloxamines (**B**). Two-dimensional sequences of Poloxamer P85 (CID: 10145203) and poloxamine 304 (CID: 86278173) were obtained from PubChem. Three-dimensional (3D) structures of the different compounds were drawn with ChemBioOffice 2012 (Chem3D Pro 13; PerkinElmer Informatics, Cambrigde, MA, USA).

Amphiphilic copolymers are particularly appealing materials due to their ability of simultaneously displaying the performance of hydrophilic and hydrophobic polymers. While the hydrophobic blocks are responsible forthe water solubility from the individualized copolymer molecules and of the creation of a suitable interface with the aqueous surrounding, the hydrophobic blocks adsorb onto hydrophobic surfaces [1]. Among the different types of amphiphilic block copolymers, those based on tri-blocks of poly-ethylene oxide (PEO, hydrophilic blocks) and poly-propylene oxide (PPO, hydrophobic blocks) are among the most widely used in pharmaceutical formulations. PEO-PPO-PEO copolymers are normally classified in two families according to the structure of their main chain. They can be linear and bifunctional tri-blocks (PEO-PPO-PEO) known as poloxamers (Pluronic^®^ or Lutrol^®^) or X-shaped (four arms PEO-PPO blocks) triblocks linked by a dyamine central core known as poloxamines (or Tetronic^®^) [1] (Figure 1). The unique structure of poloxamines confers them with multi-stimuli responsiveness. In this context, the two tertiary amine central groups play an essential role, conferring thermodynamical stability and pH sensitivity while enabling further chemical modifications in order to acquire additional properties [2]. PEO-PPO-PEO copolymers are commercially available in a broad spectrum of molecular weights (MW) and Ethylene oxide/Propylene oxide (EO/PO) ratios [3] and generally display a good cytocompatibility, without producing significant irritation after topical or parenteral administration [4,5].

The nomenclature of Pluronic^®^ includes the letters, F, P, or L, which areassociated with the physical states of these polymers, namely solid, paste and liquid, are followed by a two or three digits, which represent a numeric code related with their structural parameters [6,7]. On the other hand, Tetronic^®^-based copolymers are normally classified as a function of their hydrophilicity as highly hydrophilic (T908, T1107 andT1307), medium hydrophilic (T304, T904 and T1304)and highly hydrophobic (T701, T901, T1301, T90R4 andT150R1) [1].

### 1.1. General Aspects

A feature of PEO-PPO-PEO copolymers is the ability of the individual block copolymers or “unimers” to self-assemble into micelles in aqueous solution at concentrations higher than the critical micellar concentration (CMC). In general, the lowerthe lipophilic/hydrophilic balance (HLB) and the higher theMW, the lower the CMC found [8,9,10]. The temperature-sensitiveness of these copolymers makes the CMC decrease with the increase of temperature. In general, those copolymer varieties with longer PPO blocks require lower concentrations or temperatures for micellization to occur [9].

When the temperature and concentration increase, the micelles form 3D networks (gels) of high viscosity. Chiefly, micellization is noted at much lower concentration (<1 wt %) than those needed for gellation (>15 wt %) [8].

In general, PEO-PPO-PEO copolymers that display a sol-gel transition around 37 °C canbe orally administered or injected in the body as liquidsolutions at room temperature and, once at the physiologicaltemperature, they become semi-solid to solid gels that sustain drug release [3]. This ability of PEO-PPO-PEO copolymers to self-assemble as micelles and to undergo sol-to-gel transitions upon heating has endowed the denomination of “smart” or “intelligent” systems [11].

Even though PEO-PPO-PEO copolymers do not degrade under physiological conditions, those with molecular weights between 10 and 15 kDa are normally filtered by the kidney and cleared in urine [12]. These features make them powerful candidates to treat different human pathologies by controlling the release of different models of drugs or other bioactives or by providing a platform for the construction of biological structures acting as cell scaffolds in different tissue engineering approaches.

### 1.2. PEO-PPO-PEO-Based Micellar Systems

Polymeric micelles are nanosized carriers based on amphiphilic PEO-PPO-PEO thatcan be tailored to fit the physicochemicalcharacteristics of cargo and therapeutic requirements ofthe pathological process [13]. Structurally, polymeric micelles are based on a hydrophobic core with hydrophobic blocks that are approaching to minimize the contact with the surface, increasing the solubilization and stabilization of poorlywater-soluble drugs, and a hydrophilic shell with hydrophilic blocks in contact in the aqueous medium acting as an interface with the core as well as decreasing undesirable drug interactions with the cells [6,14]. Micellization is a process strongly driven by an entropy gain and the free energy ofmicellization is mainly a function of the PPO block [15]. Therefore PEO-PPO-PEO copolymers with larger hydrophobic domains form micelles at lower concentrations [15]. 

Polymeric micelles have a size between 10–100 nm being in the preferred range for pharmaceutical approaches [1,6]. When compared with micelles formed by common low MW surfactants, polymeric micelles have lower CMC, higher thermodynamic and kinetic stability to withstand dilution, and enhanced drugs solubilizing and stabilizing capability [16,17]. 

PEO-PPO-PEO-based micelles have been used as potential delivery systems of multiple drugs and biomolecules to increase their stability and solubility and afford protection against fast degradation [7,18,19,20,21].

### 1.3. PEO-PPO-PEO-Based Hydrogels

The viscoelastic behavior of PEO-PPO-PEO copolymers as afunction of their concentration and the effect of several stimuli such as pH (poloxamines) and temperature on their gelation properties have attracted the attention in recent yearsfordeveloping controlled delivery systems of diverse biomolecules [22]. As the temperature increases, gel systems based on PEO-PPO-PEO block copolymers become less hydrophilic due to the progressive dehydration of the polyether blocks. This fact promotes the formation of more micelles and ultimately the formation their packing into body centered cubic phase gels [23]. 

In general for a given content in EOgroups, the longer the PO block of the PEO-PPO-PEO copolymer variety, the lower thegel temperature [1]. So far, poloxamers exhibit a sol-gel transition at lower concentration compared with poloxamines. For example, while Pluronic^®^ F127 (70 wt % PEO, MW 12.6 kDa) shows a critical gel concentration around 14.8%, Tetronic^®^ 1107 (70 wt % PEO, MW 15 kDa) needs a minimal concentration around 30% to undergo sol-to-gel transition [24]. Formulation of aqueous systems based on PEO-PPO-PEO copolymers that can be easily syringeable at room temperature leading to the formation of highly viscoelastic gels at physiological temperature (37 °C) has a great potential for diverse regenerative approaches [18]. In situ gelling systems based on poloxamers or poloxamines have been studied to control the release of growth factors [25,26] and different drugs [27,28,29] and more recently for 3D cell printing applications in different tissue engineering approaches [30,31].

## 2. PEO-PPO-PEO Tri-Block Copolymers as Micellar Nanocarriers for Drug Delivery

The ability of PEO-PPO-PEO micelles to host relatively hydrophobic drugs increasing their apparent solubility and protecting them against degradation has already been reported for different models of drugs [7,32,33,34,35,36]. This solubilization capacity from poloxamers and poloxamines relies on the MW and the PEO/PPO ratio and in general the more hydrophobic the molecule, the higher solubilization extension [3]. So far, it has been reported that those copolymers with a similar HLB will display higher solubility efficiency when their MW are higher. On the other hand, a combination of poloxamers with different MW and EO/PO ratios results in mixed micelles with a superior efficiency of solubilization and higher stability upon dilution [8]. Poloxamer micelles have already been shown to increase the solubility of different models of drugs [37].

In the particular case of poloxamines, their dual responsiveness to pH and temperature and the possibility to adjust their aggregation properties by tuning the pH of the medium has attracted strong attention in recent years as a means to increase the solubility of poorly soluble drugs [1]. Chiefly, it has been reported that the higher the pH of the medium, the greater the micellization tendency and the higher the solubilization ability of poloxamines [24]. This fact has already been described for both pH-independent drugssuch asgriseofulvin [24] and efavirenz [38] and pH-dependent drugs such as triclosan [19].

## 3. PEO-PPO-PEO Tri-Block Copolymers for Passive Micellar Targeting

Direct administration of small free anticancer drugs generally results in a rapid distribution in and out of all tissues, leading to a short residence time and rapid clearance by renal filtration [39]. A valuable strength of PEO-PPO-PEO micelles is their spontaneous and preferential accumulation at biological places with vascular abnormalities leading to enhanced permeability and retention (EPR), a common fact from all tumor types except the vascular ones [3]. To take advantage of the EPR effect, the drug needs to be included into a macromolecular structure that can selectively enter the tumoral tissue through the enhanced pores and avoid its elimination by the lymphatic drainage. Generally, a more prolonged circulation time, ideally over 6 h for polymeric micelles, results in a greater possibility of reaching the cancerous tissue [40]. Polymeric micelles (10–100 nm) are large enough nanocarriers to avoid renal excretion (>50 KDa), but still small to bypass the filtration by inter-endothelial cells slits in the spleen [3]. Of further note, the hydrophilic corona from polymeric micelles plays an important role in preventing the opsonization and successive clearance by the mononuclear phagocyte system in the spleen and liver [41]. Both poloxamers and poloxamines can lead to the formation of micelles with an adequate size for the EPR effect and resist the strong dilution occurring upon contact with physiological fluids [3,10,42,43,44].

## 4. PEO-PPO-PEO Tri-Block Copolymers for Human Gene Therapy

### 4.1. Gene Transfer Vectors: Current Limitations

The identification of gene products that areinvolved in supporting the underlying cause of pathology has offered the biopharmaceutical industry an opportunity to develop compounds that can specifically target these molecules to improve therapeutic responses and lower the risk of unwanted side effects that are commonly seen in traditional small chemical-based medicines [45]. In this scenario, the development of gene delivery vehicles have emerged as a promising technology to treat different pathologies by directly transferring of genes encoding for therapeutic factors into the places of injury that result in a temporarily and spatially defined delivery of a candidate agent [46]. Current gene transfer vectors used for gene transfer of target cell populations in regenerative medicine approaches include nonviral [47] and viral vehicles [48] such as adenoviral [49], retro-/lentiviral [50,51] herpes simplex viral vectors (HSV) [52], and recombinant adeno-associated viral (rAAV) vectors [53]. Nonviral gene delivery has various advantages as it is considered a safe method that does not provoke immune responses in the host while it can be prepared in large amounts at relatively low expense [54]. While plasmid DNA (pDNA) permits transfection in vivo, packaging DNA with cationic lipids or polymers may further facilitate material uptake and transfection in vitro and in vivo [47]. Therefore, complexation of DNA with cationic polymers (polyplexes) or lipids (lipoplexes) may protect DNA against degradation by nucleases and serum components by creating a less negative surface charge and can be designed to target specific cell types through receptor-ligand interactions [55]. Yet, the main limitation of such vectors is their tendency towards aggregation and their low transfection efficiencies and short-term transgene expression levels (some days) [56]. Nonionic water-soluble polymers that do not bind or condense with DNA but significantly enhance the expression of transgenes in vitro and in vivo have noticeably received increased attention in recent years [6,57,58,59,60]. Of note, PEO-PPO-PEO copolymers based on Pluronic^®^ were reported to enhance the expression of both naked pDNA [58,60,61,62,63,64] and genes delivered using polycation-DNA complexes [65,66,67,68,69] both in vitro and in vivo. While depicting a higher efficiency, viral gene transfer is limited by the toxicity associated withsome types of vectors (adenovirus), a possible diffusion of the vectors to nontarget places, and the existence of patient-associated factors and physiological barriers (existence of neutralizing antibodies against the viral capsid, inhibition of transduction in the presence of specific anticoagulants) that may interfere with the effective delivery, processing, and expression of transgene inside the target cells [46]. The use of PEO-PPO-PEO copolymers has already been described to increase the efficiency of viral gene transfer by providing a localized delivery into the targets or protecting the vectors against physiological barriers [46], including of adenoviral [70,71,72,73,74], lentiviral [75,76], and rAAV vectors [77,78,79,80,81,82].

### 4.2. PEO-PPO-PEO Copolymers: Applications for Nonviral Gene Transfer

Skeletal muscle is a common target for gene therapy due to its accessibility and ability for production of proteins as systemic therapeutic reagents [83]. Likewise, injection of naked pDNA into muscle was already shown to provide an expression for up 19 months [84]. Still, the levels of gene expression achievable with naked DNA are often insufficient to ensure a therapeutic effect. To increase the efficiency of transgene expression, a combination of two poloxamers (SP1017: Pluronic^®^ L61 and F127) was involved to intramuscularly deliver pDNA encoding for reporter β-galactosidase gene activity (*lacZ*) in mice [58,64] (Table 1).An estimation of the levels of *lacZ* expression revealed that SP1017 considerably increased pDNA diffusion through the tissue [58,64]. A micellar solution of a PEO-PPO-PEO copolymer with an average MW of 8400 was involved as a carrier for eye-drop gene delivery of pDNA with *lacZ* in vivo [62]. Topical delivery of pDNA via PEO-PPO-PEO micelles for 48 h demonstrated *lacZ* expression was detected around theiris, sclera, conjunctiva, and lateral rectus muscle of rabbit eyes and also in the intraocular tissues of nude mice [62]. Polyelectrolyte complexes formed between DNA and polycations constitute one of the gold standards for nonviral gene delivery. So far, one of the main problems encountered using these techniques is the relatively low efficacy of DNA (or complex) release from endocytic compartments in the cytoplasm and nucleus of cells [85]. Likewise, due to charge neutralization, these complexes are often unstable in aqueous solutions and precipitate, thus hindering their application in gene delivery [65,85]. To solve these issues, a micellar solution of Pluronic^®^ P85 has been employed to deliver DNA encoding for the chloramphenicol acetyltransferase (CAT) gene complexed with poly(*N*-ethyl-4-vinylpyridiniumbromide) (PEVP) [65]. Results from this study showed an increase of CAT gene internalization and expression in different cell lines when provided via polymeric Pluronic^®^ P85 micelles [65]. Polyethyleneimine (PEI) constitutes one of the most widely used cationic polymers fornonviral gene transfer [86]. This polymer spontaneously forms interpolyelectrolyte complexes with DNA as a result of cooperative electrostatic interactions between the ammonium groups of the polycation and phosphate groups of the DNA [66]. However, one of the main drawbacks of PEI is its poor solubility upon complexation with DNA that may considerably reduce its transfection efficiency. Grafting of PEI with PEO-PPO-PEO copolymers (Pluronic^®^ P123) has been reported as a potential approach to solve such a limitation by optimizing the size of the polyplexes [67], achieving an increased expression of the reporter Firefly luciferase (*luc*) transgene, with a uniform distribution in the liver when administered intravenously in mice [66]. So far, the highest efficiency was reported to be achieved when using PEO-PPO-PEO copolymers at high HLB [68]. Similarly to PEI, polylysine (PLL) is one of the best known cationic polymers to strongly interact with pDNA, resulting in a compact polymer/DNA complex for an increased uptake of foreign genes to mammalian cells [87]. Yet, its use as a DNA carrier is still limited by its low transfection efficiency. Conjugation of PLL with Pluronic^®^ F127 showed a 2-fold increase in transfection efficiency compared with unmodified PLL [69]. Although less explored than their poloxamer counterparts, poloxamines have also been studied as integrants of scaffolds for the controlled delivery of nonviral vectors [88]. A fibrin/T904 hydrogel was synthesized to incorporate either a naked plasmid encoding for the green fluorescent protein (GFP) or polyplexe vectors based on such a reporter gene sequence. When N2A neuroblastoma cells were encapsulated in these systems, an increased transgene expression profile was noted over 2 weeks [88].

### 4.3. PEO-PPO-PEO Copolymers: Applications for Viral Gene Transfer

Development of strategies for in vivo transfer of therapeutic genes relevant to these specific vascular pathophysiologic processes offers new therapeutic possibilities for the treatment of this type of disorders. Specifically, adenoviral vectors appear to be the most efficient vectors for vascular gene transfer [70]. However, transduction of vascular vessels requires enough time (20–45 min) to permit sufficient vector uptake. Administration of adenoviral vectors with hydrogel systems based on Pluronic^®^ F127 has been reported as a potential approach to reduce the transfection time of the blood vessels with significant improvement of transgene expression both in vitro [70,71] and in vivo [72,73] (Table 2). A 10–100-fold increase on *lacZ* expression was seen upon administration of adenoviral vectors with a hydrogel system based on Pluronic^®^ F127 to vascular smooth muscle cells [70], reducing the incubation time of adenoviral vectors from 20 to 10 min without compromising transfection efficiency [71]. Intratumoral infusion of adenoviral vectors with Pluronic^®^ F127 gels was also described as a strong approach for cancer treatment by reducing the dissemination of the viral vectors from tumor to normal tissues during and after the infusion [74,77]. Pluronic^®^-based gel systems are potent adjuvants to increase the transduction efficiency of lentiviral vectors in vitro [76] and in vivo [75]. Effective transduction of astrocytes has been reported by stereotaxic delivery of a HIV-1-based lentiviral vector in a 15% PF127 gel [75]. rAAV exhibit potential advantages over other viral vectors due to their small size, ability to transduce dividing and nondividing cells, and absence of immunogenicity, constituting the most adapted vector to treat musculoskeletal disorders [89,90]. Still, although gene transfer via viral vectors is highly efficient, the existence of patient-associated factors and physiological barriers (existence of neutralizing antibodies against the viral capsid, inhibition of transduction in the presence of specific anticoagulants) may interfere with the effective delivery, processing, and transgene expression in the target cells [90]. Also, a possible dispersion of the rAAV viral particles to nontarget places may reduce their gene transfer efficiency [91]. Encapsulation of rAAV in poloxamer PF68 and poloxamine T908 polymeric micelles was shown to increase the levels of transgene expression of both mesenchymal stem cells (MSCs) [79,80] and osteoarthritic chondrocytes in vitroor in an in situ model of osteochondral defect [81,82] either with the reporter *lacZ* gene [80,81] or with highly chondrogenic genes (the sex-determining region Y-type high mobility group box 9 transcription factor—*SOX9*, the transforming growth factor beta—TGF-β) [79,82] (Table 3). Of further note, delivery of rAAV using such systems resulted in the restoration of the transduction of MSCs or chondrocytes with rAAV in conditions of gene transfer inhibition, i.e., in the presence of heparin or of a specific antibody directed against the rAAV capsid [79,81]. These features were attributed to the X-shaped structure of the poloxamines, rendering a positive charge at the physiological pH and protective effect exerted by PEO shell masking the antibody-specific rAAV capsid epitopes binding and thus the rAAV neutralization. Despite their potential advantages and simplicity, in situ gel systems based on these self-assembled copolymers remain partially hydrophilic and thus the gel depot erodes quite rapidly. To solve these drawbacks, we recently developed pseudopolyrotaxanes hydrogels by combining PEO-PPO-PEO copolymers with alpha cyclodextrins (α-CD) [92]. Results from these studies showed that incorporation of α-CD into gels based on mixtures of chondroitin sulfate (CS) or hyaluronic acid (HA) with PEO-PPO-PEO copolymer PF68 gels resulted in higher rAAV concentrations and sustained levels of transgene expression over time. Still, and while addition of α-CD resulted in the formation of more structured networks with greater elastic and viscous moduli compared with the pristine systems, a higher mechanical stability may be necessary to provide support for load-bearing functions of the cartilage tissue.

## 5. Conclusive Remarks

The application of responsive “smart” PEO-PPO-PEO polymers has a broad potential for drug delivery in different regenerative medicine approaches. While less explored, the use of these compounds as gene delivery systems represents a potential tool in gene therapy approaches. In this regard, the versatility of these molecules possibly forming micelles or gels in response to changes in temperature and/or pH makes them new gene transfer systems with a superior performance relative to the currently used gene shuttles. Diverse mechanisms have been proposed to explain the ability of these polymers to increase the levels of transgene expression. While micellar solutions from PEO-PPO-PEO copolymers were reported to increase the levels of DNA internalization [65] by interacting with the cell plasma membrane [76,93], thus overcoming potential natural barriers to vector transfection/transduction [68,79,81], gel systems were described to act as local reservoirs of vector release [70,75], increasing DNA distribution through tissues and reducing the transfection time compared with classical gene transfer vector [71]. Also, PEO-PPO-PEO copolymers have been reported to promote membrane resealing and to decrease trauma via electroporation [64,94], increasing the stability in solution of polyplexes [6,67]. There is also evidence showing that PEO-PPO-PEO copolymers may play a role in increasing transgene expression. For instance, copolymers with higher HLB are able to produce the highest improvement of gene expression levels in serum media from 10% to 50% fetal bovine serum compared with PEI-DNA complexes alone [68]. A similar trend was noted when combining such copolymers with viral vectors, promoting the highest gene transfer efficiencies when used at highly hydrophilic varieties (HLB > 24) [79]. Yet, despite potential advantages, gel systems formed by PEO-PPO-PEO copolymers are still characterized by a weak mechanical strength, a short residence time, and a high permeability which limit their applicability in gene/drug delivery [95]. Stabilization of these systems may however be promoted by crosslinking the gels [96] or by combining them with other polymers [92]. While less explored than their poloxamer counterparts, poloxamines have been also tested as gene delivery systems of both nonviral [88] and viral systems [79,81,82,92]. The X-shaped structure of these copolymers, rendering a positive charge at the physiological pH, may have an additional feature in conditions of gene transfer inhibition [79]. Altogether, the use of PEO-PPO-PEO for gene copolymers represents an emerging field in gene delivery approaches. Despite the considerable number of studies reporting the efficiency of such copolymers as adjuvants to increase the levels of transgene expression, more work is needed to elucidate their benefit-to-risk ratio for translational regenerative medicine approaches. In this scenario, limited work has been performed in relevant animal models of human diseases. Finally, and to our best knowledge, only a limited number of clinical trials reporting the application of PEO-PPO-PEO copolymers with gene transfer vectors have been reported for the treatment of intermittentclaudication in patients with moderate to severe peripheralarterialdisease [97] and as preventive vaccines against cytomegalovirus-associated disease [98].

## Figures and Tables

**Table 1:** Use of PEO-PPO-PEO copolymers for nonviral gene transfer.

Nonviral Systems	Copolymers	Genes	Targets	Administration	Observations	References
pDNA	SP1017: Pluronic^®^ L61 + F127	*lacZ*, *luc*	muscle	i.m. (rat)	10-fold increased trangene expression	[58]
		*lacZ*			increased transgene expression after electroporation	[64]
	PE6400	*lacZ*	muscle	cranial muscle (mouse)	long-term expression similar to electrotransfer	[63]
	Pluronic^®^ F68 and F127	*luc*	n.s.	in vitro BL-6 cells	increased activity in transfecting cells in the presence of 20% serum	[61]
	Pluronic^®^ P85 and L61	*luc*, GFP	n.s.	in vitro NIH3T3, C2C12 and Cl66 cells	increased transgene expression	[60]
	PEO-PPO-PEO copolymers average MW 8400	*lacZ*	Eye	ocular (rabbit, mouse)	higher transgene expression at 2 and 3 days	[62]
Polycation DNA and poly(*N*-ethyl4-vinylpyridinium)	Pluronic^®^ P85	CAT	n.s.	in vitro NIH 3T3, MDCK, and Jurkat cell lines	enhanced transfection	[65]
P123-*g*-PEI(2K)polyplexe	Pluronic^®^ P123	*luc*	n.s.	in vitro Cos-7 cells, i.v. (mouse)	more uniform distribution of transgene, significant improvement of gene expression in liver	[66]
			n.s.	in vitro prostate cancer cells (PC-3)	optimization of polyplexe size	[67]
PEI-DNA complex	Pluronic^®^ F68, F127, P105, P94, L122, L61	*lacZ*	n.s.	in vitro NIH/3T3 cells	Pluronic^®^ with higher HLB showed marked improvement of gene expression levels in serum media compared with PEI-DNA complexes alone	[68]
PEI-DNA complex or pDNA	Tetronic^®^ 904	GFP	n.s.	in vitro N2A cells	sustained transgene expression for over 2 weeks	[88]
PLL-*g*-Pluronic^®^	Pluronic^®^ F127	*lacZ*	n.s.	in vitro HeLa cells	higher transfection efficiency with polymer:DNA at 1:1	[69]

Abbreviations: pDNA: plasmid DNA; *lacZ*: *E. coli* β-galactosidase; *luc*: luciferase; PE6400: poly(ethyleneoxide)(13)-poly(propyleneoxide)(30)-poly(ethyleneoxide)(13) block copolymer; n.s.: not specified; GFP: green fluorescent protein; CAT: chloramphenicol acetyltransferase; i.m.: intramuscular; i.v.: intravenous; P123-*g*-PEI(2K)polyplexe: Pluronic^®^ 123 grafted with 2KDa polyethyleneimine; PEI-DNA complex: polyethyleneimine-DNA complex; PLL-*g*-Pluronic^®^: poly-l-lysine grafted with Pluronic^®^.

**Table 2:** Use of PEO-PPO-PEO copolymers for viral gene transfer (part I).

Viral Systems	Copolymers	Genes	Targets	Administration	Observations	References
Adenovirus	Pluronic^®^ F127	*lacZ*	cardiovascular	in vitrovascular smooth muscle cells	high pericellular concentrations of vector and 10- to 100-fold increase of transduction	[70]
	Pluronic^®^ F127	*lacZ*	vascular	in vitrovascular smooth muscle cells; in vivo balloon injured carotid arteries (rat)	improved gene transfer efficiencies	[71]
	Pluronic^®^ F127	*lacZ*, *luc*	vascular	in vivo percutaneous administration in iliac arteries (rabbit)	increased efficacy of percutaneous gene transfer and reduced transfection time	[72]
	Pluronic^®^ F127	*gax*	vascular	in vivo external iliac artery with channel balloon catheter	*gax* overexpression inhibits neointimal hyperplasia and lumen loss in atheromatous stented rabbit iliac arteries	[73]
	Pluronic^®^ F127	GFP, *luc*	solid tumors	in vivo intratumoral infusion (mouse)	blocked convection of viral vectors in the interstitial space and the lumen of microvessels in the vicinity of the infusion site	[74]
Lentivirus	Pluronic^®^ F127	GFP	CNS	in vivo injection to the thalamus (rat)	increased transduction of astrocytes at injection site	[75]
	Pluronic^®^ F108	GFP, *luc*	n.s.	in vitro HEK293T, KARPAS-299, SUDHL-1, SR-786, SUP-M2, and PANC-1 cell lines	specific contribution to efficiency of each adjuvant; polybrene: charge protector and poloxamer synperonic F108: membrane modulator	[76]

Abbreviations: *lacZ*: *E. coli* β-galactosidase; *luc*: luciferase; *gax*: growth arrest homeobox; GFP: green fluorescent protein; rAAV: recombinant adeno-associated viral vectors; CNS: central nervous system; DC: dendritic cells; n.s.: not specified.

**Table 3:** Use of PEO-PPO-PEO copolymers for viral gene transfer (part II).

Viral Systems	Copolymers	Genes	Targets	Administration	Observations	References
rAAV	Pluronic^®^ F127	GM-CSF	solid tumors	in vivo intratumoral infusion (mouse)	higher efficiency by combining DC, local tumor irradiation and controlled supply of recombinant mGM-CSF with Pluronic^®^	[77]
	Pluronic^®^ F68	*lacZ*	adipose tissue	in vivo inguinal (mouse)	increased transgene expression after 4 weeks	[78]
	Pluronic^®^ F127	*lacZ*	cartilage	in vitro hMSCs	controlled release of rAAV for high efficiencies over time and gene expression levels similar to those achieved by direct vector application	[80]
	Pluronic^®^ F68, Tetronic^®^ 908	RFP, *lacZ*, and *SOX9*	cartilage	in vitro hMSCs	encapsulation of rAAV in polymeric micelles for effective, durable, and safe modification of hMSCs; restoration of hMSC transduction in conditions of gene transfer inhibition; effective chondrogenesis	[79]
	Pluronic^®^ F68, Tetronic^®^ 908	*lacZ*	cartilage	in vitro hOACs in situ human osteochondral model	micellar encapsulation for increased stability and bioactivity of rAAV; high levels of safe transgene expression in vitro and in experimental osteochondral defects in situ	[81]
	Pluronic^®^ F68, Tetronic^®^ 908	TGF-β	cartilage	in vitro hOACs in situ human osteochondral model	increased levels of transgene expression compared with free vector treatment; high proteoglycan deposition and increased cell numbers in hOACs in vitro; high deposition of type-II collagen and reduced hypertrophy in osteochondral defects models in situ	[82]
	Pluronic^®^ F68, Tetronic^®^ 908	*lacZ*	cartilage	in vitro hMSCs	high concentrations of rAAV; sustained levels of transgene expression over time	[92]

Abbreviations: rAAV: recombinant adeno-associated viral vectors; *lacZ*: *E. coli* β-galactosidase; GM-CSF: granulocyte-macrophage colony-stimulating factor; hMSCs: human mesenchymal stem cells; RFP: red fluorescent protein; *SOX9*: sex-determining region Y-type high mobility group box 9; hOACs: human osteochondral chondrocytes; TGF-β: transforming growth factor beta.
